# Experimental Sleep Deprivation Results in Diminished Perceptual Stability Independently of Psychosis Proneness

**DOI:** 10.3390/brainsci12101338

**Published:** 2022-10-03

**Authors:** Leonie J. T. Balter, Granville J. Matheson, Tina Sundelin, Philipp Sterzer, Predrag Petrovic, John Axelsson

**Affiliations:** 1 Department of Clinical Neuroscience, Karolinska Institutet, 171 77 Stockholm, Sweden; 2 Stress Research Institute, Department of Psychology, Stockholm University, 106 91 Stockholm, Sweden; 3 Molecular Imaging and Neuropathology Division, Department of Psychiatry, Columbia University, New York, NY 10032, USA; 4 Department of Biostatistics, Mailman School of Public Health, Columbia University Mailman School of Public Health, New York, NY 10032, USA; 5 University Psychiatric Clinics Basel, University of Basel, 4002 Basel, Switzerland; 6 Center for Cognitive and Computational Psychiatry, Department of Clinical Neuroscience, Karolinska Institutet, 171 77 Stockholm, Sweden; 7 Center for Psychiatric Research, Department of Clinical Neuroscience, Karolinska Institutet, 171 77 Stockholm, Sweden

**Keywords:** perceptual stability, predictive processing, psychosis proneness, sleep deprivation, random dot kinematogram

## Abstract

Psychotic disorders as well as psychosis proneness in the general population have been associated with perceptual instability, suggesting weakened predictive processing. Sleep disturbances play a prominent role in psychosis and schizophrenia, but it is unclear whether perceptual stability diminishes with sleep deprivation, and whether the effects of sleep deprivation differ as a function of psychosis proneness. In the current study, we aimed to clarify this matter. In this preregistered study, 146 participants successfully completed an intermittent version of the random dot kinematogram (RDK) task and the 21-item Peters Delusion Inventory (PDI-21) to assess perceptual stability and psychosis proneness, respectively. Participants were randomized to sleep either as normal (8 to 9 h in bed) (*n* = 72; *M*_age_ = 24.7, *SD* = 6.2, 41 women) or to stay awake through the night (*n* = 74; *M*_age_ = 24.8, *SD* = 5.1, 44 women). Sleep deprivation resulted in diminished perceptual stability, as well as in decreases in perceptual stability over the course of the task. However, we did not observe any association between perceptual stability and PDI-21 scores, nor a tendency for individuals with higher PDI-21 scores to be more vulnerable to sleep-deprivation-induced decreases in perceptual stability. The present study suggests a compromised predictive processing system in the brain after sleep deprivation, but variation in psychosis trait is not related to greater vulnerability to sleep deprivation in our dataset. Further studies in risk groups and patients with psychosis are needed to evaluate whether sleep loss plays a role in the occurrence of objectively measured perceptual-related clinical symptoms.

## 1. Introduction

The brain has an extraordinary capacity to create stable interpretations of noisy or ambiguous signals in an attempt to yield reliable information from our ever-changing external world. However, variability in this capacity is observed across various states and conditions. For example, in psychotic states perceptions are often anomalous, manifested in hallucinations, delusions, and perceptual deficits [1,2,3,4]. Perceptions of form, size, and color may change when experiencing the external world, suggesting an instability in sensory processing. Likewise, sleep loss is associated with instabilities in a range of perceptual experiences such as visual distortions, illusions, and somatosensory changes [5].

How the brain creates stable perceptual experiences is not entirely known, but one possible explanation comes from the theory of hierarchical predictive processing [6]. This model proposes that incoming signals will be compared with an expectation (often denoted a prior), a process which takes place already in low-level perceptual (i.e., visual, auditory) networks. When there is a mismatch between incoming information and the prior, an error signal will be forwarded to the next hierarchical level, where a similar comparison between the higher order prior and the input (error) signal will take place. As this higher-level prior has more degrees of freedom it also has a larger possibility to explain and thereby reduce the error signal. In this way error signals are forwarded and mitigated in the hierarchical information processing stream, where higher hierarchical levels are more concerned with global features while low-level systems handle perceptual details. Arguably, this hierarchical processing helps create stable perceptual states.

It has been proposed that imprecision in low-level priors can cause perceptual instability and psychosis [7,8,9,10]. The reason for such imprecise low-level priors in psychosis-associated phenotypes is not known but may relate to aberrant salience caused by dopaminergic dysfunction [7,11] or to an excitatory-inhibitory imbalance relating to glutamatergic dysfunction [12].

Distortion of perceptual experiences that occur after sleep loss have many similarities to those found in patients with psychosis. Severe sleep loss can cause healthy individuals to experience temporary perceptual distortions of stimulus intensity, quality, or form [5]. A gradual progression from blurred vision and diplopia to visual distortions and illusions, and finally hallucinations, has been reported with increasing time awake, pointing to a gradual degradation of perceptual processes with sleep loss [5]. The underlying mechanisms of information-processing dysfunction after sleep loss remain unclear, but may be related to the presence of “local sleep” during wake. Sleep can occur locally in regional networks or individual neurons, which is related to attenuated or delayed responses as well as cognitive lapses [13,14,15,16]. Other mechanisms that might explain an increase in variability of information processing after sleep loss include local extracellular increases of inhibiting metabolites such as adenosine [17], reduced activity in arousal-promoting systems [18], and reduced functional connectivity between prefrontal cognitive control regions and other parts of the brain [19]. Together, these mechanisms may explain findings of increased signal variability, both locally and globally, in sleep-deprived subjects [20,21]. Although not discussed in detail previously, these mechanisms may interfere with predictive processing of information throughout the brain and cause more imprecise low-level priors. This would, in effect, cause a similar low-level information processing disturbance as in psychosis.

The relationship between sleep and psychosis is further emphasized by the clinical observation that sleep disturbances are associated with more severe psychotic symptoms in patients with schizophrenia spectrum disorders. Sleep disturbances also predict transition from at-risk to first-episode psychosis [22,23,24]. A similar relationship between sleep disturbances and psychotic symptoms such as hallucinatory experiences has been suggested in healthy individuals with high levels of psychosis proneness [25]. Psychosis proneness may be defined as a personality trait distributed within the normal population showing different degrees of non-clinical psychotic symptoms including hallucinations and delusions [26]. These data suggest a prominent role for sleep behind psychotic symptoms. However, it is unclear whether perceptual instability, i.e., the tendency to switch between alternative perceptual states when confronted with ambiguous sensory information, worsens after acute sleep loss in individuals with higher psychosis proneness. Consistent with the vulnerability-stress model, it is expected that those with an underlying vulnerability (e.g., high psychosis proneness) would be more sensitive to the effects of a stressor (e.g., sleep deprivation) than those with less vulnerability (e.g., low psychosis proneness) [27].

Studies using questionnaires or interviews indicate altered perception after sleep loss [5]. While perception is foremost a subjective experience, symptom self-reports present phenotypical endpoints that provide little information on underlying processes and subtle aspects of perception. Moreover, self-reports on interoceptive experiences are malleable to metacognitive capacity and therefore confounded. A powerful tool to assess perceptual instability is through ambiguous stimuli such as random-dot kinematograms (RDK) that are compatible with two mutually exclusive perceptual interpretations. The ambiguity maximizes the need for perceptual inference and consequently involves endogenous predictions [28,29]. Studies using ambiguous RDKs have shown that psychosis-related states such as schizophrenia and delusion proneness exhibit reduced perceptual stability, which has been interpreted as a consequence of imprecise low-level priors [2,8,9,29].

In the current preregistered study, we assessed the hypothesis that healthy individuals undergoing experimental sleep deprivation (versus normal sleep) show weakened low-level priors, resulting in diminished perceptual stability, as assessed using the RDK task. We further hypothesized that individuals higher in psychosis proneness would show a larger perceptual instability, and that this effect would be stronger in the presence of sleep deprivation (i.e., an interaction effect). The Peters Delusions Inventory-21 (PDI-21) [30] was used as a proxy for psychosis proneness.

## 2. Materials and Methods

### 2.1. Participants

One hundred and eighty-two healthy individuals participated in the current study (mean age = 25.4, *SD* = 6.5, range 18–45, *n* = 103 women), of whom 178 contributed valid data for the PDI-21 and 150 for the RDK task. As described in the preregistration, individuals for whom no response was provided for over 60% of their RDK trials were excluded, as well as individuals who exhibited fewer than two changes of direction over the entire course of the task. Of the remaining 146 participants included in the final analysis, 72 were part of the normal sleep group (mean age = 24.7, *SD* = 6.2, 41 women) and 74 were part of the sleep deprivation group (mean age = 24.8, *SD* = 5.1, 44 women). Data were collected continuously between February 2015 and May 2016. Potential participants were recruited via posters and online advertisement. An online screening was completed with the following exclusion criteria (see also [31]): sleep problems (e.g., disturbed sleep, difficulties falling asleep, difficulties waking up, light or shallow sleep) rated as one to three times per week or more during the last six months, general poor sleep quality (rated as “quite bad” or “very bad”), intake of medication or supplements in order to sleep better, poor habitual sleep (having less than six hours of sleep four times per week or more), a sleep need outside of 7–9 h per night, health problems (e.g., psychiatric problems such as depression, anxiety, worry, pain in the chest or back, diabetes) for which a doctor was contacted during the last year, a night-shift within three weeks prior to the testing day, consuming more than four cups of coffee per day, being a current smoker, or an age outside the age-range requirement of 18–45 years. All subjects gave written informed consent and received financial compensation for participation (non-sleep-deprived, 800 Swedish krona; sleep-deprived, 1500 Swedish krona). For other findings from this study, see [31,32,33,34,35]. All procedures were approved by the Stockholm Regional Ethical Review Board in Stockholm (no. 2014/1766-32).

### 2.2. General Procedures

Following screening, participants completed the PDI-21 [28] and were instructed to have three consecutive nights of 8 to 9 h in bed per night. After these three nights, participants were pseudorandomized to either sleep at home for one more night of 8 to 9 h in bed (turn off the lights at 23:00 ± 60 min, get up at 07:00 ± 60 min; normal-sleep group) or to come to the laboratory for a night of sleep deprivation (sleep-deprivation group). Pseudorandomization was performed between the normal sleep and sleep-deprived groups, ensuring a relatively similar distribution of age and men and women in the two sleep conditions. On the penultimate day, participants were informed by telephone during lunchtime whether they were in the normal-sleep or sleep-deprived group. Each participant was instructed to keep a daily sleep diary for three days before the test day and wear an actigraph (GeneActiv Sleep, Activinsights, Kimbolton, UK, or MotionWatch 8, CamNtech, Cambridge, UK) on their non-dominant wrist. Participants were asked to avoid naps, abstain from alcohol, and not drink caffeinated drinks later than the morning of the day prior to testing.

Participants in the sleep-deprivation condition arrived at the laboratory at 22:00 and were monitored throughout the night. During this time, they were free to choose their activities (e.g., study, use their mobile phone, or watch a film) and were kept in a light-controlled room. The experimenter was present at all times. Low-sugar snacks were provided if the participant was hungry and a 15 min morning walk was taken to reduce the difference in light exposure and activity compared to what participants in the normal-sleep group may experience while travelling from their home to the laboratory. The normal-sleep group came to the laboratory at 10:00 the following morning. A detailed description of the sleep protocol has also been described elsewhere (e.g., [32,35]). In the afternoon after the final sleep schedule, participants completed the random dot kinematogram (RDK) (starting between 15:45 and 16:45). 

### 2.3. Materials

#### 2.3.1. Psychosis Proneness

Participants completed the Swedish translation of the Peters Delusion Inventory-21 (PDI-21), a 21-item questionnaire designed to measure individual variation in delusional ideation in the general population [28]. The questions in the PDI-21 asses a variety of delusional beliefs with “yes-or-no questions”. For example: “Do you ever feel as if things in magazines or on TV were written especially for you?”; “Do you ever feel as if there is a conspiracy against you?”. If the subject responds “yes”, different dimensions are rated for each statement (i.e., distress, preoccupation, and conviction, rated on a 1 to 5 scale with higher scores indicating more distress, preoccupation, or conviction, respectively). As described in our preregistration (https://aspredicted.org/OLN_COH, accessed on 1 September 2022), individuals lacking more than 25% of the items within the PDI-21 scale would be excluded (*n* = 4). Individuals who did not complete one or more items had their scores imputed as the average of their remaining questions. In total, there were 178 valid completions of the PDI-21 scale, after excluding individuals whose scales were incorrectly completed. The primary measure of interest, as defined in the preregistration, was the total conviction score (i.e., the sum of all conviction subscale ratings), since it has been shown to relate to RDK survival probability [9]. Endorsed items (questions answered with “yes”) were rated from 1 = “don’t believe it is true” to 5 = “believe it is absolutely true” and thus the total conviction score can range from 0 (none of the items were endorsed) to a maximum of 105 (all items were endorsed and rated with 5). We also defined the mean distress score, rated from 1 = “not at all distressing” to 5 = “very distressing”, as a secondary measure of interest, i.e., the mean of all endorsed distress subscale ratings. The distress subscale score can range from 0 to a maximum of 5. The difference between mean and sum scores is that the former is aimed at capturing the salience of the subscale of the beliefs, while the latter captures a combination of the number and salience of those beliefs. These results are shown in the Supplementary Materials. Several tertiary measures of interest were defined in the preregistration document, but we decided a priori that we would only examine those which showed a Pearson correlation coefficient *r* <= 0.5 with either of the measures from the primary and secondary analyses.

#### 2.3.2. Perceptual Stability

**Figure 1:**
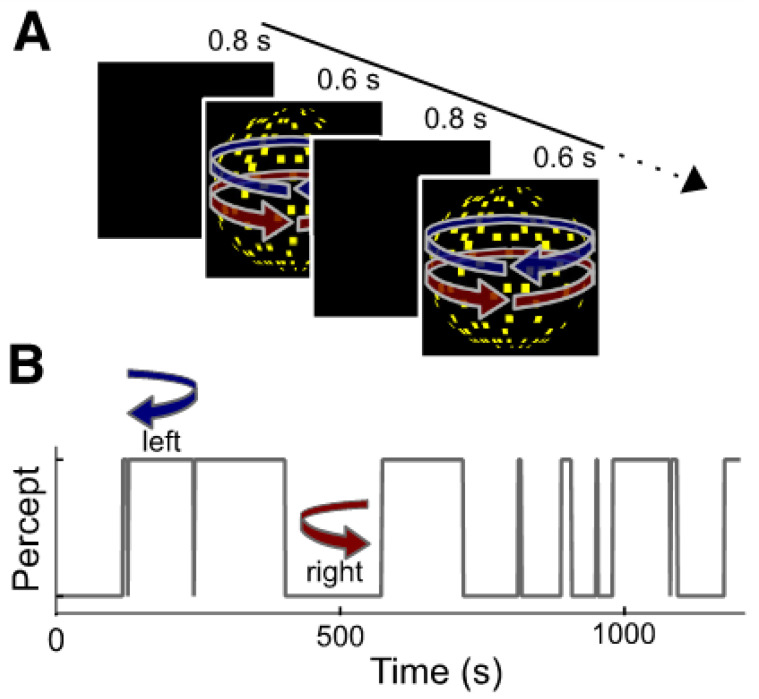
(**A**) A typical trial of the Random Dot Kinematogram (RDK) task. Ambiguous dot kinematogram are presented repeatedly for 600 ms interleaved by a blank screen of 800 ms duration. The sphere can be perceived as rotating either leftward or rightward. Participants are asked to report the perceived direction of the rotation by making a button press. (**B**) The perceptual time course from an example participant. The stabilizing effect of endogenous predictions are automatically built up during intermittent presentation of the ambiguous stimulus, this results in participants tending to have the same percept across successive presentation cycles. The figure is replicated with permission from Schmack et al. [9].

We used a 10-minute-long presentation of the intermittent random-dot kinematogram (RDK) to assess perceptual stability as described in Schmack et al. [9]. Figure 1 displays a trial of the RDK task. In brief, the task involved 430 presentations of a 3-D sphere stimulus made up of 450 randomly distributed yellow squared “dots”. Half of the dots moved coherently leftward and the other half of the dots moved coherently rightward on a black background with a central fixation cross. The 3-D sphere was framed by a white square. The RDK gives rise to the perception of a sphere rotating in depth. It is perceptually ambiguous with respect to the rotation direction, thus resulting in bistable perception with alternations between the two possible perceptual states over prolonged viewing. At each presentation, participants reported via a button press whether they perceived the sphere as turning to the left or to the right. Each stimulus was presented for 600 ms, interleaved by blank screens with an 800 ms duration. As described in the preregistration, individuals for whom no response was provided for over 60% of their trials were excluded, as were individuals who exhibited fewer than two changes of direction over the entire course of the task. Not providing a response does not necessarily mean that participants were not attending to the task: rather, some participants perceived the sphere as moving in both directions at once for some trials, and were instructed not to answer either direction for these trials. In total, 150 participants had valid RDK outcomes. The final dataset included 54,673 trials with valid responses, of which participants reported 4758 changes in direction.

### 2.4. Modeling and Statistical Analysis

A binomial regression model was used. To this end, every trial of each individual was modeled as an event in which participants could choose either the same direction as in the trial preceding it, or a change in direction. A higher survival probability, is considered to indicate greater perceptual stability at the individual level, reflecting stronger sensory predictions/priors. To test the hypothesis that sleep deprivation would result in diminished perceptual stability, i.e., a lower survival probability, a binary predictor of sleep deprivation was included in the model. Additionally, to assess whether greater psychosis proneness would be associated with diminished perceptual stability, which would be most apparent following sleep deprivation, both a main effect of psychosis proneness, as well as an interaction between sleep deprivation and psychosis proneness were tested. As described in the preregistration document, we also identified that there might be a need to account for the effects of directional preference as an additional covariate. The analysis was defined as a hierarchical binomial model with individual as a random effect. Frequentist analysis was performed using the *lme4* package [36], and Bayesian analysis was performed using STAN [37] and the *brms* package [38] in R [39]. Bayes Factors were calculated using Savage-Dickey ratios [40], from 16,000 post-warmup samples. For directional hypotheses, only the samples in the hypothesized direction were used for the estimation of Bayes Factors in order to test the directional hypotheses.

Priors over the covariates representing the hypothesized predictors were defined in the preregistration as being normal zero-centered priors with a *SD* equal to a change of 5% following sleep deprivation and a 10% change for delusional ideation over the range of 75% of the PDI-21 scores. For the mean survival probability, the mean survival probability in Schmack et al. [9] was approximately equal to 91%, and equal to 94% when PDI-21 scores were estimated to be zero. In Schmack et al. [29], the survival probability had a mean of approximately 96%. For this reason, we defined a prior for the survival probability centered at 94% from Schmack et al. [9], with one standard deviation from the mean spanning from 90% to 96%. The resulting prior was a normal distribution centered at 2.75 with a *SD* of 0.55 in the space of the logit link function. Using this mean, we defined a regularizing prior for sleep deprivation with a mean of zero and a *SD* of 0.66 to represent the expected 5% change in survival probability. We also used this same prior for the effect of directional preference, but without a directional specificity. For the PDI-21 scales, we defined zero-centered priors with a *SD* of 0.022 for every unit increase in total conviction score, and 0.46 for every unit increase in mean distress score, to represent a 10% change in survival probability over 75% of the potential range of each scale as was preregistered.

### 2.5. Changes to Preregistered Analysis Plan

During modeling, we identified the influence of several additional factors which we did not anticipate during our preregistration; these were included in the final model. Firstly, we included a covariate for the trial number as a proportion of the total number of trials, in order to accommodate a gradual change in performance over the trial period. Secondly, we included the possibility of an interaction between trial number and sleep deprivation, as the change in performance over the ten minutes could be greater in sleep-deprived individuals. Lastly, there was a tendency for individuals to experience direction changes following missed trials so we defined an additional covariate for the previous trial having been missed. To further assess the influence of missing responses, we ran additional frequentist analyses estimating the number of trials responded to for which the previous response was missing, with a random effect of individual, predicted by sleep deprivation, as well as sleep deprivation and PDI-21 conviction scores. All additional covariates were defined with bidirectional normal priors with the same *SD* as that for sleep deprivation.

## 3. Results

### 3.1. Summary Statistics

#### 3.1.1. Trait Levels of Psychosis Proneness (PDI-21)

Endorsed Yes/No items ranged from 0 to 17 (*M* = 4.5, *Mdn* = 4.0, *SD* = 3.4) out of a total of 21, total conviction scores ranged from 0 to 54 (*M* = 12.4, *Mdn* = 10.5, *SD* = 10.3). The results using the secondary outcome of the PDI-21, i.e., the mean distress score, are presented in the Supplementary Materials for transparency reasons. All tertiary measurements described in the preregistration were correlated with an *r* > 0.5 with total conviction scores or mean distress scores, resulting in their exclusion as a priori decided upon and described in the preregistration document. Correlations between all outcomes of the PDI-21 questionnaire can be found in Supplementary Figure S2.

#### 3.1.2. Perceptual Stability (RDK)

There was a preference for judging the dots to rotate in a left direction (64% of all trials). We therefore included directional preference in the final model.

#### 3.1.3. Data Exclusions

PDI-21 data were excluded for four individuals owing to invalid completions of the PDI-21. RDK data were excluded for six individuals owing to an excessive number of missed trials, four of whom were sleep deprived. RDK data were also excluded for an additional 20 measurements who experienced fewer than two changes of direction. Of this group, six were sleep-deprived, and the mean PDI-21 score was 4.75 which was not significantly different from the mean score in the remainder of the sample with valid PDI-21 measurements (*M* = 4.4, *t*_26.2_ = −0.48, *p* = 0.64).

### 3.2. Modeling

Results for the primary PDI-21 measure of interest, the total conviction score, yielded highly similar results to that of the secondary measure of interest. For this reason, all results from the other covariates are presented from the model with the primary measure of interest. The full results with the secondary outcome measure can be found in Supplementary Materials.

**Figure 2:**
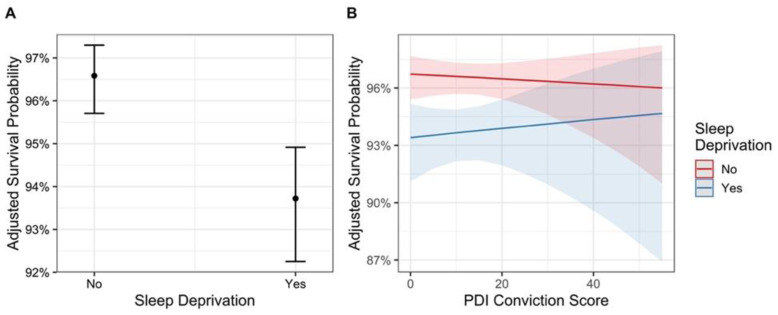
(**A**) Conditional effects of sleep condition (sleep deprivation no/yes) on adjusted survival probability and (**B**) interaction effect of sleep condition (sleep deprivation no (“normal sleep” group) in red and sleep deprivation yes (“sleep deprived” group) in blue) by PDI-21 conviction score. Error bars and bands represent 95% confidence intervals.

**Figure 3:**
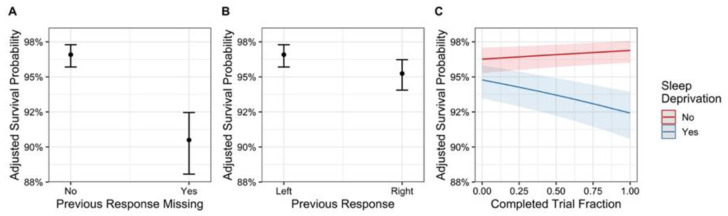
(**A**) Condition effects of previous response missing, (**B**) previous response left or right, and (**C**) interaction effect of sleep condition (sleep deprivation no (“normal sleep” group) in red and sleep deprivation yes (“sleep deprived” group) in blue) by completed trial fraction. Error bars and bands represent 95% confidence intervals.

There was moderate evidence for a main effect of sleep deprivation, showing that sleep deprivation resulted in a lower survival probability (BF_10_ = 4.3, *p* = 0.033, Figure 2A). There was no substantive evidence for an increase in survival probability with trial number after normal sleep (though with a marginally significant *p* value) (main effect, BF_10_ = 1.3, *p* = 0.026, Figure 3C), however strong evidence for a decrease in survival probability with trial number following sleep deprivation (interaction effect: BF_10_ > 1000, *p* < 0.001, Figure 3C).

We observed no evidence for main effects or interaction effects with sleep deprivation for the PDI-21 scores. For total conviction scores, the null model was slightly preferred over the alternative hypothesis for both the main effect of conviction score (BF_01_ = 1.7, *p* = 0.440), as well as its interaction between conviction score and sleep deprivation (BF_01_ = 2.8, *p* = 0.295, Figure 2B), although both of these comparisons exhibit only weak evidence in favor of the null hypothesis owing to their low BFs.

Lastly, strong evidence was observed for missing the previous trial (BF_10_ > 1000, *p* < 0.001, Figure 3A) and for the direction with a preference for rotating to the left (BF_10_ > 1000, *p* < 0.001, Figure 3B). Sleep deprivation was associated with a significant increase in trials for which the previous response was missing, with estimates of 2.5% following normal sleep, and 8.0% following sleep deprivation (*p* < 0.001). PDI-21 scores, however, were not associated with a significant change in previously missed responses after correction for sleep deprivation (*p* = 0.89).

## 4. Discussion

In the current preregistered study, we examined whether healthy individuals show diminished perceptual stability after sleep deprivation, and whether psychosis proneness was related to increased vulnerability to sleep-loss-induced perceptual instability. In this sample of healthy individuals, sleep deprivation diminished perceptual stability, suggesting weakened low-level priors, particularly at later stages of the task. However, psychosis proneness was not associated with perceptual stability and the effect of sleep deprivation on perceptual stability was not modulated by psychosis proneness. The finding that sleep deprivation causes perception to become instable is consistent with a meta-analysis by Waters et al. [5] suggesting that visual perception, conceptualized as visual distortions, illusions, and hallucinations, is affected by increased time awake. While many previous studies used subjective reports [5], here a more objective test was used, in the form of RDK, that confirms an effect of lack of sleep on low-level perceptual processing. Perceptual stability measured with RDK has previously been used as an index of strength in low-level priors [8,9]. Thus, one interpretation of the results is that sleep loss has an impeding effect on the strength of low-level priors. In other words, sleep loss directly affects predictive processing.

A question that arises is how sleep loss may affect predictive coding. The worsening in perceptual stability after sleep deprivation, and its worsening over time, could be due to slower processing or local sleep in early sensory areas, leading to weaker low-level priors locally in affected brain areas. This would be in line with sleep loss typically causing a deterioration of performance across time due to intrusions of slower processing speed, attention lapses [41,42], or sleep occurring in areas being used for extensive time periods [15]. This corresponds with our results showing that sleep loss resulted in diminished perceptual stability both directly as well as indirectly through an increase in the number of missed responses, resulting in an even greater combined effect than either of their conditional effects. Furthermore, because of the shortened 10-minute RDK task relative to previous studies [9], the majority of individuals excluded as a result of excessively high survival probability, i.e., ceiling effects, were not sleep-deprived – implying that the true effect of sleep deprivation may be even greater than could be shown in the analysis. Another neural mechanism by which sleep deprivation may affect perceptual stability is through a gradual decrease in stability of connectivity between prefrontal and visual areas. Indeed, numerous studies have shown that sleep deprivation predominantly affects prefrontal cortex functioning [43,44,45], as well as visual areas [15,46,47]. This could lead to disturbances of higher-level visual processing. Finally, local sleep occurs frequently after sleep loss throughout the brain [13,14,15,16] and may also occur in visual processing streams, affecting processing stability.

Previous studies show that both patients with schizophrenia and healthy individuals with high psychosis proneness (specifically conviction scores) exhibit weaker perceptual stability as measured with RDK (e.g., [9,29]). We therefore hypothesized that the effects of sleep deprivation on perceptual stability would be more pronounced in participants with higher psychosis proneness. However, our study does not support this hypothesis as we did not find a relation between perceptual stability and psychosis proneness nor an interaction between sleep loss and psychosis proneness on perceptual stability. The reason for this lack of effect may be that our sample was overall more healthy than previous samples, having relatively low psychosis proneness scores and low variability (our sample had a PDI-21 mean of 4.5 and *SD* of 3.4, compared to the original study by Peters et al. [28] which had a mean of 6.7 with a *SD* of 4.4). A reason for this difference may be that the individuals included in the present study were carefully screened for various psychiatric disorders and unhealthy sleep, which are often present in psychosis-associated phenotypes [25,48]. This poses several possibilities, including that sleep disturbances would be less problematic for individuals low in psychosis proneness, or that sleep deprivation could explain part of the relationship between high psychosis proneness and a weaker perceptual stability found in previous studies. It may thus be relevant to study whether unhealthy sleep is a driving factor for acute perceptual problems in high-risk groups and patients, or alternatively is a risk factor for developing new delusional beliefs that become stable across time.

The present study has a number of limitations, strengths, and potential avenues for future research. The study should be seen as large scale in the field of sleep deprivation, including 182 subjects (*n* = 146 in the final analyses), and with a rather strong manipulation of sleep loss (no sleep for a whole night as compared to normal sleep). Despite the large-scale approach, with a focus on individual differences and clear impairments seen for several other cognitive functions [32,33], the study had relatively low power for making inferences on anything but medium or large effect sizes. Use of a within-subject design is a potential approach to reduce the potential influence of differences between groups and to improve power. There is also a need to explore how other types of unhealthy sleep, such as chronic sleep restriction or clinical sleep disturbances, influence perceptual stability. Moreover, the study results are limited to healthy young individuals. The test was carried out in the late afternoon in all subjects, a time where many cognitive functions show a performance dip [33], and it would be of high relevance to study whether other factors such as light exposure or caffeine can ameliorate or worsen perceptual stability. Future studies could also assess the neural mechanisms by which sleep deprivation may affect perceptual stability, e.g., whether local sleep in visual processing streams occurs after sleep deprivation, which can impact predictive processing. The missing responses observed in the current study could be due to intrusions of local sleep [16], however to confirm this, measures such as intracranial EEG are needed, something that was not included in the present study.

## 5. Conclusions

In conclusion, our study shows that sleep deprivation reduced perceptual stability, but no evidence was found for individuals with higher subclinical psychosis to be more susceptible to the effects of sleep deprivation on perceptual stability. The present study thus suggests a compromised predictive processing system in the brain after sleep loss. The underlying neural mechanism and the relation to psychosis proneness and clinical psychosis need to be further investigated.

## Data Availability

The data and code will be made publicly available upon publication in the Open Science Framework (OSF) and a link will be provided.

## Supplementary Materials

The following supporting information can be downloaded at: https://www.mdpi.com/article/10.3390/brainsci12101338/s1, Table S1. Model coefficients for the primary model; Table S2. Model coefficients for the secondary model; Figure S1. Interaction effect of sleep condition by PDI-21 mean distress score. Error bars and bands represent 95% confidence intervals; Figure S2. Correlations between all outcomes of the PDI-21 questionnaire. Kernel density plots of the distributions of each outcome are presented along the diagonal.
