# Supplementary material for: Experimental Sleep Deprivation Results in Diminished Perceptual Stability Independently of Psychosis Proneness
Source: Brain Sci. 2022 Oct 3;12(10):1338. doi: 10.3390/brainsci12101338 (PMC9599202; doi:10.3390/brainsci12101338)
Supplement: Supplementary file 1 [file brainsci-12-01338-s001.zip › brainsci-1903178-supplementary.pdf]

SUPPLEMENTARY MATERIALS

Secondary and tertiary measures of interest

Mean distress scores ranged from 0 to 4.2 (M = 1.7, Mdn = 1.7, SD = 0.9). The correlation between total conviction scores and mean distress scores was r=0.48. None of the tertiary measures of interest were examined as they were all correlated at r>0.5 with either total conviction scores or mean distress scores.

Results were highly similar for the models including the primary and secondary PDI-21 outcomes of interest. The full model outcomes for the Bayesian models are presented below for both. For mean distress scores, the null and hypothesized effects were equally likely for the main effect (BF_{10} = 1.04, p = .094), but the null model was preferred for the interaction between mean distress scores and sleep deprivation (BF_{10} = 4.5, p = .201) (see Figure S1).

Table S1. Model coefficients for the primary model

	Survival	
Predictors	*Odds Ratios*	*CI (95%)*
Intercept	27.09	18.76 – 38.66
Sleep Deprivation	0.64	0.41 – 0.99
Previous Response L/R	0.71	0.66 – 0.76
Trial Proportion	1.20	1.02 – 1.42
Previous Response Missing	0.34	0.31 – 0.37
PDI-21 Conviction Total	1.00	0.98 – 1.02
Sleep Deprivation : Trial Proportion	0.56	0.45 – 0.70
Sleep Deprivation : PDI-21 Conviction Total	1.01	0.98 – 1.03
**Random Effects**		
σ^2	3.29	
$\tau_{00\ ID}$	1.04	
ICC	0.24	
N_{ID}	146	
Observations	54673	

Table S2. Model coefficients for the secondary model

	Survival	
Predictors	*Odds Ratios*	*CI (95%)*
Intercept	33.73	20.37 – 56.31
Sleep Deprivation	0.57	0.30 – 1.07
Previous Response L/R	0.71	0.66 – 0.76
Trial Proportion	1.20	1.02 – 1.43
Previous Response Missing	0.34	0.31 – 0.37
PDI-21 Distress Mean	0.86	0.67 – 1.10
Sleep Deprivation : Trial Proportion	0.56	0.45 – 0.70
Sleep Deprivation : PDI-21 Distress Mean	1.13	0.80 – 1.58
**Random Effects**		
σ^2	3.29	
$\tau_{00\ ID}$	1.03	
ICC	0.24	
N_{ID}	146	
Observations	54673	

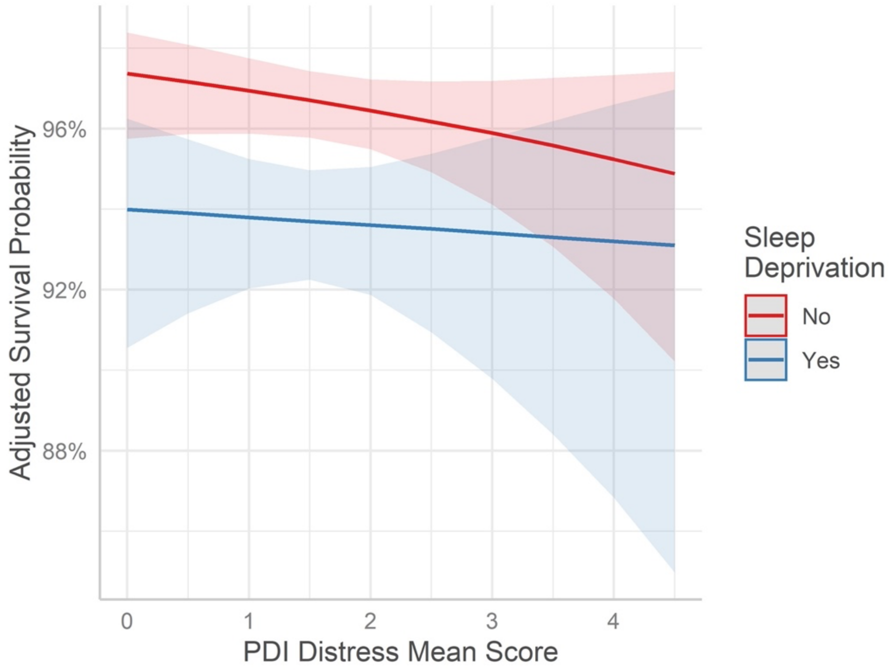

Figure S1. Interaction effect of sleep condition by PDI-21 mean distress score. Error bars and bands represent 95% confidence intervals

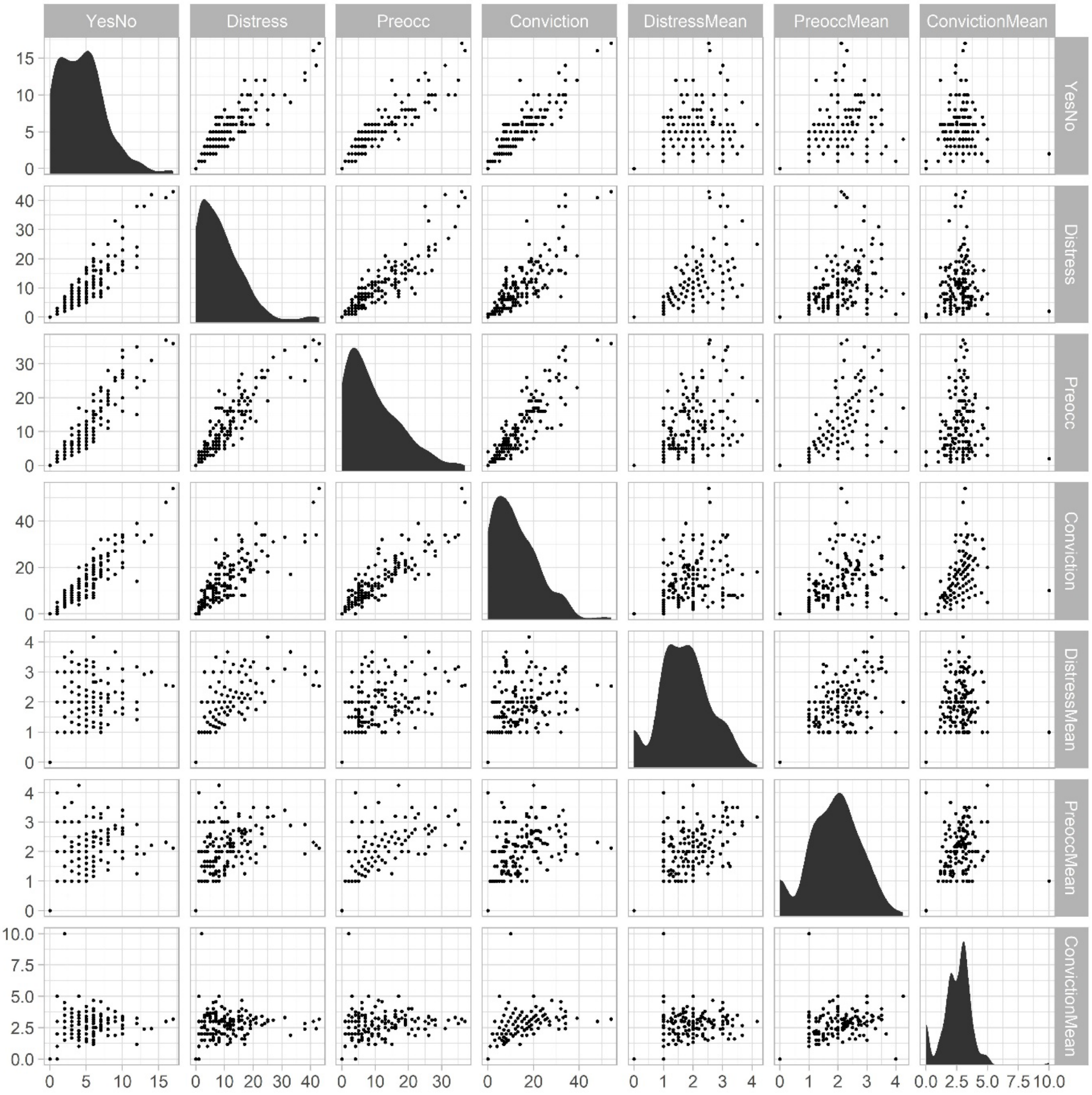

Figure S2. Correlations between all outcomes of the PDI-21 questionnaire. Kernel density plots of the distributions of each outcome are presented along the diagonal
